# Prospects for elimination of soil-transmitted helminths

**DOI:** 10.1097/QCO.0000000000000395

**Published:** 2017-10

**Authors:** Kristjana H. A´sbjo¨rnsdo´ttir, Arianna R. Means, Marleen Werkman, Judd L. Walson

**Affiliations:** a DeWorm3, The Natural History Museum, London, UK; b Department of Global Health, University of Washington, Seattle, Washington, USA and; c Department of Infectious Disease Epidemiology, London Centre for Neglected Tropical Disease Research (LCNTDR), St. Mary’s Campus, Imperial College London, London, UK

**Keywords:** disease elimination, soil-transmitted helminths, transmission interruption

## Abstract

**Purpose of review:**

Soil-transmitted helminths (STH) are endemic in 120 countries and are associated with substantial morbidity and loss of economic productivity. Although current WHO guidelines focus on morbidity control through mass drug administration (MDA), there is global interest in whether a strategy targeting disease elimination might be feasible in some settings. This review summarizes the prospects for switching from control to an elimination strategy.

**Recent findings:**

STH control efforts have reduced the intensity of infections in targeted populations with associated reductions in morbidity. However, adults are not frequently targeted and remain important reservoirs for reinfection of treated children. Recent modeling suggests that transmission interruption may be possible through expanded community-wide delivery of MDA, the feasibility of which has been demonstrated by other programs. However, these models suggest that high levels of coverage and compliance must be achieved. Potential challenges include the risk of prematurely dismantling STH programs and the potential increased risk of antihelminthic resistance.

**Summary:**

Elimination of STH may offer an opportunity to eliminate substantial STH-related morbidity while reducing resource needs of neglected tropical disease programs. Evidence from large community trials is needed to determine the feasibility of interrupting the transmission of STH in some geographic settings.

## Introduction

Soil-transmitted helminths (STH) are a group of neglected tropical diseases (NTDs) that include hookworm (Necator americanus and Ancylostoma duodenale), roundworm (Ascaris lumbricoides), and whipworm (Trichuris trichiura). STH are endemic in at least 120 countries and are estimated to account for over 5 million disability-adjusted life years (DALY) [1■,^2^] and substantial productivity loss [3,4] in endemic countries. The WHO Roadmap for NTDs [5] and 2012 London Declaration on Neglected Tropical Diseases [6] focus on the control of STH morbidity through mass drug administration (MDA) to school-age (SAC) and preschool-age (PSAC) children. There has been increasing interest in moving beyond morbidity control toward elimination of many NTDs, including STH [7 – 10]. We review recent literature relevant to the prospects for switching from a control to an elimination strategy for STH.

## Soil-Transmitted Helminths Morbidity Control

The current WHO endorsed strategy for the control of STH aims to eliminate STH as a public health problem, defined by the WHO as a reduction in prevalence to less than 1% of moderate or high intensity infection [11]. This target is based on evidence that severity of STH-associated morbidities is highly associated with an individual’s intensity of infection. STH infections of moderate-to-high intensity are associated with diarrhea, anemia, chronic inflammation, and malnutrition and with disrupted growth and cognitive impairment in children [12,13^&^].

Key Points

- Mathematical modeling indicates that interrupting the transmission of STH may be possible through MDA of albendazole expanded from child-targeted to community wide, even without universal access to water and sanitation.
- New opportunities to pursue transmission interruption are emerging because of pursuit of elimination strategies for other neglected tropical diseases, demonstrated feasibility of community-wide MDA, reductions in prevalence of STH through current morbidity control efforts, and advances in molecular diagnostics.
- Challenges to an MDA-based STH transmission interruption strategy include the need for high coverage and compliance, the definition and accurate determination of interruption status, and the potentialfor anthelminthic resistance.
- A switch from morbidity control to a transmission interruption strategy for STH has risks, including the emergence of resistance to antihelminthics and the loss of resources for morbidity control in the event that elimination is unsuccessful.

Given that the diagnosis of STH infection requires laboratory capacity and skilled microscopists and that treatment with a single dose of albendazole or mebendazole is inexpensive, well tolerated, and can be delivered to high-risk groups by nonmedical personnel in schools or communities, the WHO recommends presumptive deworming through MDA in endemic areas [14]. Although there is ongoing debate about the economic, cognitive, and morbidity impact at the population level of this strategy [13^&^,^15^,^16^,^17^^&^,^18^], it can effectively eliminate infections of intensities understood to cause morbidity [19^&^,^20^^&^]. Specifically, current WHO guidelines focus on routine empiric deworming of all SAC, PSAC, and women of childbearing age without reliance on diagnostic testing of individuals before treatment [11]. The WHO recommends deworming of SAC annually in areas where pre-MDA prevalence is between 20 and 50%, and twice annually where pre-MDA prevalence is greater than 50% [5].

The WHO NTD Roadmap has set a target of achieving 75% coverage of SAC and PSAC in all endemic countries by 2020 [5]. The success of the this ambitious strategy in treated populations is evident [14,21,22]: despite recent coverage estimates suggesting that only about half of all populations in need of treatment are being treated, global prevalence and intensity of infection in children have decreased significantly and school-based deworming has resulted in substantial reductions in STH-associated morbidity in many settings [2,19^&^,^23^].

## The Case For Community-Wide Mass Drug Administration For Soil-Transmitted Helminths

Despite these gains, pediatric reinfection rates post-treatment are high [24,25^&^,^26^], and school-based deworming has limited impact on overall community-wide prevalence [27] and intensity [28] of infection. When left untreated, adults continue to serve as reservoirs of STH infection in the community, ensuring continued reinfection of treated children, and sustaining transmission [29]. This is especially true in communities where hookworm is the predominant infection, as prevalence of hookworm infection peaks in adulthood [30]. Modeling of STH transmission under repeated rounds of MDA suggests that due to continued transmission at the community level, PSAC-targeted and SAC-targeted deworming programs will need to continue indefinitely – or at least until economic development, access to adequate water and sanitation, and other socio demographic changes occur to maintain benefit [10,31].

Expanding treatment with MDA to all individuals in a community has been shown to result in greater reductions in STH prevalence, even among children, than SAC-targeted and PSAC-targeted MDA [32^&^]. Models (and some empiric data) suggest that trans-mission interruption may be possible through chemotherapy alone, provided that the treated population is expanded to all age groups and high coverage is achieved [8 – 10,31,33,34]. Experience from the Global Alliance to Eliminate Lymphatic Filariasis, which provides community-wide MDA with a package of drugs, including albendazole, has demonstrated that achieving high treatment cover-age through community delivery of MDA is possible [35]. In fact, a substantial proportion of albendazole treatment worldwide is currently provided by MDA programs targeting lymphatic filariasis (LF), not by STH programs [9]. However, as LF programs achieve successful LF elimination and transition to post-MDA surveillance, community-wide MDA through the LF platform will cease. As a result, many populations in formerly LF and STH coendemic areas stand to lose the benefit to adults as well as the indirect benefit to children of community-wide treatment; and an estimated 14% of children are at risk of losing cover-age altogether, as SAC-targeted and PSAC-targeted programs are not in place in all STH endemic areas covered by the LF program [36^&^].

## Water, Sanitation, And Hygiene

Although current WHO goals emphasize morbidity control, case studies of successful interruption of STH transmission do exist. STH were previously highly prevalent in the southeastern United States, South Korea, and Japan, where sustained control efforts through large-scale screening and MDA appear to have interrupted transmission [37 – 39]. It is important to note that though these successful programs relied heavily on mass chemotherapy, each of them took place during a time of significant economic development and improvements in access to water, sanitation, and hygiene (WASH). Improvements in WASH are critical from a human rights perspective [40] and have been advanced as crucial to the control or elimination of STH [41,42,43^&^]. However, the evidence is mixed regarding the influence of WASH resources on STH prevalence and infection intensity [43^&^,^44^^&^,^45^,^46^^&&^, 47,48]. The impact of WASH is influenced by the specific intervention(s) used, the quality of the intervention, consistent WASH usage, and other individual-level behaviors, as well as contributors to the environmental reservoir such as WASH usage and behaviors of other members of the community,[44^&^] agricultural activities [49,50], and environmental factors such as precipitation and soil composition [43^&^,^51^,^52^]. Successful WASH interventions are not only challenging to systematically measure but also costly to implement relative to MDA, so are rarely provided at sufficient scale; universal access remains a distant target [40,53]. Most available data on the effect of WASH interventions on STH prevalence or incidence come from observational and/or cross-sectional studies and there is limited high-quality trial evidence [54], although several randomized trials are ongoing or results pending publication [55].

## Theoretical Possibilities: What Do The Models Tell Us?

Models of STH transmission under repeated rounds of community-wide MDA suggest that transmission interruption in a variety of transmission settings may be possible through chemotherapy alone [31,34]. STH species cannot auto infect, meaning that successful reproduction requires at least one male and one female worm within a single human host [56]. At very low prevalence and mean intensity of infection, there is low likelihood of a single host being infected with both sexes. Therefore, it is not necessary for MDA to continue until all worms are eliminated [57]. The dynamics of STH infection in a population are defined by three parameters: the endemic prevalence that existed prior to the initiation of MDA, extinction of the parasites, and an unstable breakpoint – a theoretical threshold at which MDA may be ceased and where prevalence, rather than increasing until reaching equilibrium at pre-MDA levels, will instead decline over time and eventually reach extinction [58^&^]. The breakpoint of transmission defines the moment at which reproduction cannot occur any longer and the helminth population collapses without further treatment of remaining infections.

Mathematical models can simulate this behavior and investigate the impact of MDA on the likelihood of transmission interruption. These models estimate the feasibility of transmission interruption and the frequency and the number of rounds of MDA required to achieve it. Key parameters include the dominant species of STH in each setting; the pre-MDA transmission intensity (R_0_) for the dominant species; the age profile of infection; effective cover-age of MDA, a combination of coverage, compliance, and drug efficacy; and immigration of new hosts and parasites into the community being treated [31,34,57,58^&^,^59^^&^]. Each presents challenges for application to the real-world setting. Species distribution has implications for the persistence of infectious material in the environment [57], the age distribution of hosts and the importance of expanding chemotherapy to include adults [60], and the efficacy of each round of albendazole [61^&&^]. Pre-MDA data on species-specific STH prevalence are often unavailable, of low quality, or limited to SAC, whereas estimating pre-MDA transmission intensity based on post-MDA prevalence data introduces additional uncertainty [62^&&^].

Despite these challenges, models of STH trans-mission and the impact of repeated rounds of MDA have been compared and validated against declines in prevalence observed in real-world settings [59^&^,^63^^&^]. Two models fitted to the same baseline data obtained from a study of community-wide treatment in India – one a deterministic and the other a stochastic model – predicted the short-term impact of deworming with comparable accuracy and largely agreed on longer term predictions including the potential for transmission interruption despite differences in methodology [59^&^].

## Challenges To Interruption Through Mass Drug Administration Alone

There are several foreseeable challenges and risks to the success of an MDA-based transmission interruption strategy for STH. Even in moderate transmission intensity settings, models predict that high coverage and compliance, perhaps as high as 90% for SAC and PSAC and 80% for adults, are needed to achieve treatment interruption with MDA alone [56,58^&^]. Achievement of high coverage in other NTD elimination campaigns, including in ongoing LF programs, is encouraging; however, compliance with offered treatment is equally important [64^&^,^65^^&^]. Estimates of compliance with MDA for helminths range from 19.5 to 99% [66^&^] and highlight the need for more consistent definitions and longitudinal studies of compliance and cover-age when MDA is delivered to communities and directly observed therapy may not always be feasible. In addition, systematic nonparticipation in MDA, in which some individuals are less likely to be offered [67,68] or to accept [69,70] MDA – as opposed to random distribution of noncompliance at a given coverage level – is particularly challenging, increasing the predicted number of rounds of MDA required to interrupt transmission even at levels of noncompliance less than 10% [58^&^]. Non-compliance may increase with treatment fatigue in the community, particularly as morbidity, and perceived need for intervention is reduced.

Although models can provide estimates of the coverage and number of rounds needed to break transmission in a given setting, in practice, deter-mining whether transmission has been interrupted presents a significant logistical challenge. The achievement of interruption can only be verified by removing drug pressure and ceasing MDA for a predefined period to monitor for recrudescence [58^&^]. However, ceasing MDA activities in resource-limited settings carries a risk that program resources will be diverted elsewhere and programs difficult to restart in the event that recrudescence is observed [9]. For this reason, a threshold must be selected to optimally distinguish between areas where transmission has been interrupted and areas with continued transmission with a high positive predictive value (PPV) [58^&^]. In this context, PPV is defined as the proportion of communities below the threshold in which prevalence will continue to de-cline toward elimination rather than bouncing back to pre-MDA levels. Assessment should ideally be timed so that programmed MDA activities can go forward in the event that a community does not meet the threshold; however, PPV is improved the longer the interval between MDA and assessment [58^&^,^71^]. Modeling to compare several thresholds suggests that absolute prevalence, rather than change in prevalence or a prevalence ratio, best discriminates between communities that proceed to elimination and those that bounce back, and that a posttreatment prevalence of less than or equal to 2% has a PPV more than 80% for most pretreatment transmission intensity scenarios [71,72].

Another logistic consideration is how to accurately determine when the absolute prevalence reaches the threshold with a high PPV for transmission interruption. Microscopy-based diagnostic methods are not sensitive at low intensity [73,74] and given that prevalence of infection is highly correlated with infection intensity, mean intensity is expected to be extremely low as populations approach the transmission breakpoint [2] and current microscopic methods would likely be inadequate for the documentation of transmission interruption. Although the assessment of novel diagnostics for STH is somewhat hampered by the lack of a gold standard [75], in field-based studies, quantitative polymerase chain reaction (qPCR) consistently detects more infections than microscopy-based methods. Using PCR as a pseudogold standard, these studies have found sensitivity of two-stool two-slide Kato-Katz as low as 70% for Ascaris and 32% for N. americanus relative to multi-parallel qPCR [73], whereas sensitivity of sodium nitrate flotation was 83% for Ascaris and 34% for hookworm relative to multiplex PCR [73,76]. Detection of DNA by qPCR is possible at concentrations as low as 1 fg/μl [77] and qPCR appears to have superior sensitivity [78^&&^] at known concentrations of eggs corresponding to low-to-moderate intensity of infection, as well as reduced variability [79] when compared with Kato-Katz. Recent advances in molecular diagnostics, including qPCR, may meet the necessary performance characteristics to be useful for the documentation of an absolute prevalence 2% or less.

Transmission interruption through MDA relies on the continued efficacy of benzimidazoles. Al-though the cure rate of a single dose of benzimidazoles is as high as 97% for Ascaris [80], the efficacy against hookworm is variable and may be linked to nutritional status [25^&^,^26^]. Albendazole alone has a low cure rate for *Trichuris* infection [80,81] and MDA has only a moderate impact on mean infection intensity [82], suggesting that transmission interruption in areas where Trichuris is dominant may require the use of combination therapy [25^&^,^61^^&&^].

The potential emergence of resistance to benzimidazoles is a major concern when expanding MDA to the whole community [83,84] particularly given the lack of availability of second-line treatment options and the potential for the success of the STH morbidity control strategy to be undermined. Anthelmintic resistance, including multidrug resistance [85], is a well documented issue in animal populations [86] and some mutations associated with benzimidazole resistance have been identified in eggs from human stool [87]. Although any large-scale MDA with benzimidazoles may risk producing resistance [54], in animal husbandry, strategies to combat resistance include the exclusion of a subset of the population from deworming to maintain refugia [88,89]. The exclusion of adults from MDA mirrors this strategy in human populations, suggesting that expansion of MDA to include all age groups risks accelerating the emergence of resistance unless transmission is interrupted within a fairly small number of rounds.

## Conclusion

Interrupting the transmission of STH in some geo-graphic areas could theoretically eliminate substantial morbidity and productivity loss while also the reducing resource burden that STH programs demand. Transmission interruption would eliminate the need for continued MDA, freeing up resources for other public health activities in resource-limited settings and limiting the need for pharmaceutical company donation programs which are currently planned through 2020. An elimination strategy through MDA only, while requiring substantial in-vestment, is nevertheless potentially more achievable in many settings than universal WASH improvement. However, there are many foreseeable challenges to such a strategy, and most evidence for its feasibility comes from mathematical models. Although these provide crucial information to in-form a roadmap of the strategy and its requirements, given the risks to existing morbidity control efforts, assumptions must be tested in well conducted population-based trials before the merits of such a strategic shift can be determined.
